# Comparative Safety and Effectiveness of Heterologous CoronaVac–ChAdOx1 versus Homologous CoronaVac Vaccination in a Real-World Setting: A Retrospective Cohort Study

**DOI:** 10.3390/vaccines11091458

**Published:** 2023-09-05

**Authors:** Ayakarn Ruenkham, Suriyon Uitrakul, Peninnah Oberdorfer, Siriporn Okonogi, Wasan Katip

**Affiliations:** 1 Department of Pharmacy, Srisangwan Hospital, Mae Hong Son 58000, Thailand; 2 Department of Pharmaceutical Care, School of Pharmacy, Walailak University, Nakhon Si Thammarat 80160, Thailand; suriyon.ui@wu.ac.th; 3 Epidemiology Research Group of Infectious Disease (ERGID), Chiang Mai University, Chiang Mai 50200, Thailand; aoberdor@med.cmu.ac.th; 4 Division of Infectious Diseases, Department of Pediatrics, Faculty of Medicine, Chiang Mai University, Chiang Mai 50200, Thailand; 5 Department of Pharmaceutical Sciences, Faculty of Pharmacy, Chiang Mai University, Chiang Mai 50200, Thailand; okng2000@gmail.com; 6 Center of Excellence in Pharmaceutical Nanotechnology, Faculty of Pharmacy, Chiang Mai University, Chiang Mai 50200, Thailand; 7 Department of Pharmaceutical Care, Faculty of Pharmacy, Chiang Mai University, Chiang Mai 50200, Thailand

**Keywords:** heterologous vaccine regimen, CoronaVac, ChAdOx1, real-world setting, homologous vaccine regimen

## Abstract

**Background**: The severe acute respiratory syndrome coronavirus 2 (SARS-CoV-2) pandemic has outpaced vaccine availability and delivery from vaccine manufacturers, and thus, a scarcity of vaccines happened to many countries around the world. In Thailand, the mixing of different types of vaccines was approved and clinically implemented partially due to concerns about the availability and efficacy of one vaccine. **Objective**: This study aimed to investigate the effectiveness and safety of heterologous CoronaVac–ChAdOx1 nCoV-19 vaccines compared with the usual regimen of homologous CoronaVac–CoronaVac. A retrospective cohort study was conducted by dividing patients into the CoronaVac–CoronaVac group and the CoronaVac–ChAdOx1 group. **Results**: A total of 875 patients received vaccinations at Srisangwan Hospital between April to October 2021 and were included for analysis. The patients in both homologous and heterologous groups had low rates of COVID-19 infection. In addition, the hospitalization rates in the 40 days after the second vaccination were low in both regimens. Minimal adverse events (AE) were reported in both groups, including local AE (e.g., discomfort at the injection site, rash, soreness, swelling, and redness) and systemic AE (e.g., fever, headache, weariness, nausea, vomiting, diarrhoea, and myalgia). Moreover, several factors were associated with lower adverse events following immunization (AEFIs), including age ≥ 50 years, male, and body weight ≥ 50 kg. In contrast, thyroid disease, diabetes mellitus, allergic rhinitis, and psychiatric disorders were independent risk factors associated with an increase in AEFIs. **Conclusions**: The heterologous CoronaVac–ChAdOx1 and homologous CoronaVac–CoronaVac regimens were promising vaccination strategies for the prevention of SARS-CoV-2 infection. However, the heterologous CoronaVac–ChAdOx1 potentially caused fewer AEFIs compared with the homologous CoronaVac–CoronaVac regimen.

## 1. Introduction

The most significant scientific breakthrough in the fight against the severe acute respiratory syndrome coronavirus 2 (SARS-CoV-2) pandemic was the speed of vaccine creation. Despite the fact that the efficacy and safety of all approved vaccines were demonstrated in many large clinical trials, post-marketing surveillance study in a larger population in routine clinical practice has recently been considered necessary [1]. The rapid escalation rate of COVID-19 has outstripped vaccine availability and delivery time [2,3], and thus, the availability of vaccines, especially in Thailand and other less industrialized countries, usually relies on the government’s capability.

The first type of COVID-19 vaccine that was approved by the Thai Food and Drug Administration (FDA) and used in Thailand was CoronaVac from CoronaVac Life Sciences, Beijing, China. It was developed using inactivated SARS-CoV-2 and was approved for people aged 18 to 59 years. A clinical study involving over 10 million Chilean people demonstrated 65.9% effectiveness after receiving two doses of CoronaVac [3]. The latter type of vaccine approved by the Thai FDA is ChAdOx1 nCoV-19 vaccine from AstraZeneca, Oxford, United Kingdom. Unlike CoronaVac, this vaccine was developed using an adenoviral vector encoding SARS-CoV-2 spike (S) protein, which was shown to elicit a substantial immune response in several phase II/III trials [4,5,6].

At the time that the ChAdOx1 vaccine was available in Thailand, CoronaVac had been distributed to a large number of Thai people and became the fundamental vaccine in the country. Once the ChAdOx1 vaccine was rolled out, the majority of Thai people got their first inoculation with CoronaVac and therefore had to choose the second dose of vaccine from either CoronaVac or ChAdOx1. Based on the information available at that time, the Thai Advisory Committee on Immunization Practice recommended administering the second dose of ChAdOx1 following the first dose of CoronaVac, especially for individuals with severe comorbid conditions or those who experienced adverse events following immunization (AEFIs). These adverse events included body rashes, allergies, and vaccination-stress-related responses that required hospitalization after receiving the CoronaVac vaccine [7]. Moreover, this recommendation was informed by immunogenicity data from Yorsaeng et al. [8], who found that the heterologous CoronaVac–ChAdOx1 regimen produced antibody levels comparable with those resulting from two doses of ChAdOx1. As a result, in July 2021, the heterologous regimen was proposed in the national vaccination programme in Thailand, permitting a 3- to 4-week interval between the first dose of CoronaVac and the second dose of ChAdOx1 nCoV-19 [7,8].

One of the benefits of the heterologous CoronaVac–ChAdOx1-S regimen is the shorter interval compared with two doses of ChAdOx1-S; the waiting time can be reduced from 10 weeks to 4 weeks with similar antibody levels [9,10]. The immunogenicity and adverse effects between heterologous the CoronaVac–ChAdOx1 regimen and the homologous CoronaVac and homologous ChAdOx1 regimens were reported in a pilot study in 2021 [11]; 354 participants were recruited to four vaccination groups: CoronaVac-ChAdOx1 group (n = 155), homologous CoronaVac group (n = 32), homologous ChAdOx1 group (n = 47), and COVID-19 patients (n = 120). The amount of IgG antibodies against the receptor-binding domain (anti-SRBD) in the CoronaVac–ChAdOx1 group after the second booster dose at 2 weeks was higher than at 4 weeks. The anti-SRBD level in the CoronaVac–ChAdOx1 group was significantly greater at 4 weeks following the second booster dosage than in the homologous CoronaVac, homologous ChAdOx1, and control groups (*p* < 0.001) [11]. These results were similar to the study of Wanlapakorn et al. [9]; the heterologous CoronaVac–ChAdOx1 regimen generated stronger SARS-CoV-2 RBD-specific antibody responses and neutralizing activity against the wild type and variants of concern than the licensed CoronaVac–CoronaVac vaccine schedule. However, despite these results, the effectiveness and adverse events (AEs) in a larger number of samples remain limited, emphasizing the urgent need for more data to support vaccination recommendations [8].

One of the major concerns about heterologous vaccination regimens, such as CoronaVac–ChAdOx1 or ChAdOx1–mRNA vaccines is the safety profile. Although reported AEs were not often and mainly not serious [1,12], the interaction between two different types of the vaccine was still unknown. Therefore, this study aimed to compare the effectiveness, safety, and risk factors associated with the AEFIs of homologous and heterologous types of vaccine administered to healthy individuals; the homologous regimen was defined as two doses of CoronaVac, and the heterologous regimen was defined as the first dose of CoronaVac followed by a ChAdOx1 dose.

## 2. Materials and Methods

A retrospective cohort study was conducted at Srisangwan Hospital, which is a secondary hospital in Maehongson province, Thailand, from April 2021 to October 2021 during the period of the Delta variant COVID-19 pandemic. Due to the significant developments and changes in vaccine availability, as well as the government strategies during the pandemic, the data in this study were obtained from April to October 2021 to emphasize the unique dynamics and challenges of that period in Thailand. This study was approved by the ethics committee on human research of the Maehongson Provincial Public Health Office in Maehongson province (MSH REC-001.2565) with a waiver of informed consent for retrospective data collection under the condition of anonymously stored data collected. All methods were performed in accordance with the relevant guidelines and regulations.

### 2.1. Participants and Treatment

Those eligible for inclusion were people older than or equal to 18 years old of age who received both the first and second doses of the COVID-19 vaccine at Srisangwan Hospital between April and October 2021; only people with the first dose of the CoronaVac vaccine were included. The information on recruited people was collected from the Srisangwan Hospital database.

Immunization regimens for all people were selected depending on the availability of vaccines at that time, together with the official recommendation of the Thai Advisory Committee on Immunization Practice; therefore, there were two groups of people in this study. The first group received the CoronaVac vaccine as a first dose and the CoronaVac vaccine three weeks after as a second dose. The other received the CoronaVac vaccine as a first dose but received a second dose of the ChAdOx1 vaccine three weeks after the first dose.

### 2.2. Data Collection

Information was gathered from the recruited population using several approaches. General characteristics, including age, sex, drug allergy history, comorbid diseases, and the interval between first and second doses, were recorded from the patient database of the hospital. The incidence of COVID-19-positive cases and hospitalizations due to COVID-19 was monitored from day 0 (14 days after the second inoculation) up to day 40.

For the safety profiles, there were two surveillance systems used in this study. First, active AEFI surveillance was performed in the hospital by healthcare professionals. People who received any vaccine had to stay in the hospital for 30–60 min to observe any acute adverse effects that might happen. Furthermore, if there was no reported adverse effect in the hospital, they were asked to self-report any adverse effect that might occur at home via a mobile application called ‘Mo Prom’. Mo Prom is a national AEFI surveillance application that reminds an individual to self-report any delayed adverse effect on days 1, 7, and 30 after inoculation. This study gathered information on adverse effects from both reports by healthcare professionals and people themselves via the application. The safety information after the first and second doses of vaccination was analyzed in this study.

The adverse effects collected in this study were classified as AEFIs. They included the information as follows:

- The number of AEFIs.
- Age and gender differences in the distribution of AEFIs.
- The effects of AEFIs: the various symptoms or adverse events that people may encounter after being vaccinated.
- Interventions taken to deal with AEFIs.
- Seriousness of AEFIs defined by the World Health Organisation, which include one or more of the following factors: hospitalization, life-threatening, disability, congenital anomaly/birth defect, and death.
- Local AEs, systemic AEs, and vital sign severity of AEFIs:
  (a) Local AEs: these refer to side effects or reactions that occur primarily at the site where the vaccine was administered.
  (b) Systemic AEs: systemic AEs are side effects or reactions that affect the entire body rather than just the injection site.
  (c) Vital sign severity of AEFIs: This refers to the assessment of the severity of AEFIs based on vital signs, which are critical measurements of a person’s body functions. Vital signs include parameters such as body temperature, heart rate, blood pressure, and respiratory rate.
- Causality of AEFIs defined by the World Health Organization: The determination or assessment of whether a given adverse event is caused by the administration of a vaccine. The WHO’s causality assessment typically involves the following categories: certain, probable, possible, unrelated, and indeterminate.
- AEs of special interest (AESIs), such as myocarditis and anaphylactic shock.

### 2.3. Statistical Analysis

Descriptive statistics were used to describe general characteristics and basic information of people in the two groups, i.e., homologous (CoronaVac–CoronaVac) and heterologous (CoronaVac–ChAdOx1) first and second doses. The chi-squared test or the Fisher’s exact test (if less than five observations) for categorical data was performed to compare basic characteristics, while either an independent T-test or Mann–Whitney U test was used depending on a normal distribution of the data. A significance level of 0.05 was set for all analyses.

Due to an imbalance in baseline characteristics between the treatment groups, an inverse-probability-weighted (IPW) propensity score adjustment was performed to reduce potential bias. This technique is widely used to minimize the potential of confounding factors between treatment arms. The propensity score was calculated using multivariable logistic regression. The variables included in the propensity score calculation were body weight, hypertension, age, diabetes mellitus, history of drug allergy, and allergic rhinitis. The variables were selected for analysis based on their association with outcomes of interest and baseline covariates with *p*-values of <0.20.

The Cox proportional hazards regression model was used to investigate the effect of time on outcomes. Also, univariate Cox regression analysis was used to investigate the variables associated with AEFIs. The adjusted hazard ratios (HRs) and 95% confidence intervals (CIs) of related factors were evaluated by a full model of Cox proportional hazards regression analysis (inverse probability weighting using the propensity score for baseline covariate adjustment). A 2-sided α of 0.05 was considered statistically significant for all analyses. We calculated the incidence rates of AEFIs per 1000 people within 30 days after vaccination. The data analysis was performed using Stata software, version 14 (Stata Corp, College Station, TX, USA).

## 3. Results

### 3.1. Baseline Characteristics

**Figure 1:**
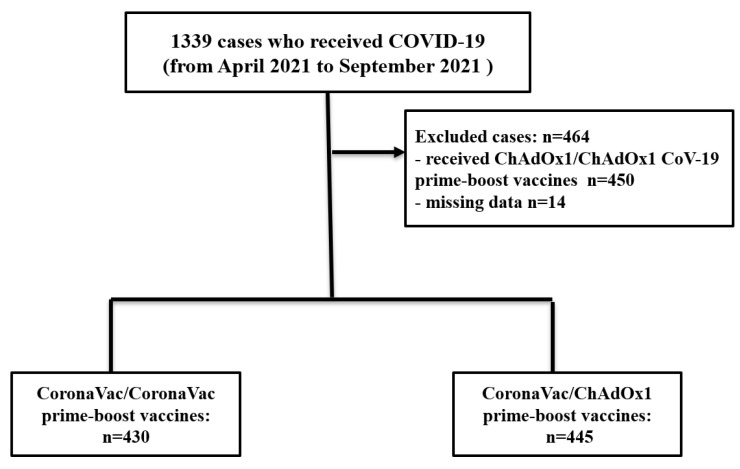
Flowchart of participants in this study.

A total of 1339 participants who received the COVID-19 vaccine at the hospital were screened, and 464 of them were excluded because they received ChAdOx1–ChAdOx1 vaccines (n = 450) and because of incomplete data (n = 14). Of all 875 participants analyzed, 430 participants received homologous CoronaVac–CoronaVac and 445 participants received heterologous CoronaVac–ChAdOx1 vaccines (Figure 1). The mean age of the homologous CoronaVac–CoronaVac group was 40.20 ± 10.01 years and of the CoronaVac–ChAdOx1 group was 41.83 ± 14.03 years. The summary of characteristics of people receiving the COVID-19 regimens is presented in Table 1.

### 3.2. Effectiveness and Safety

**Figure 2:**
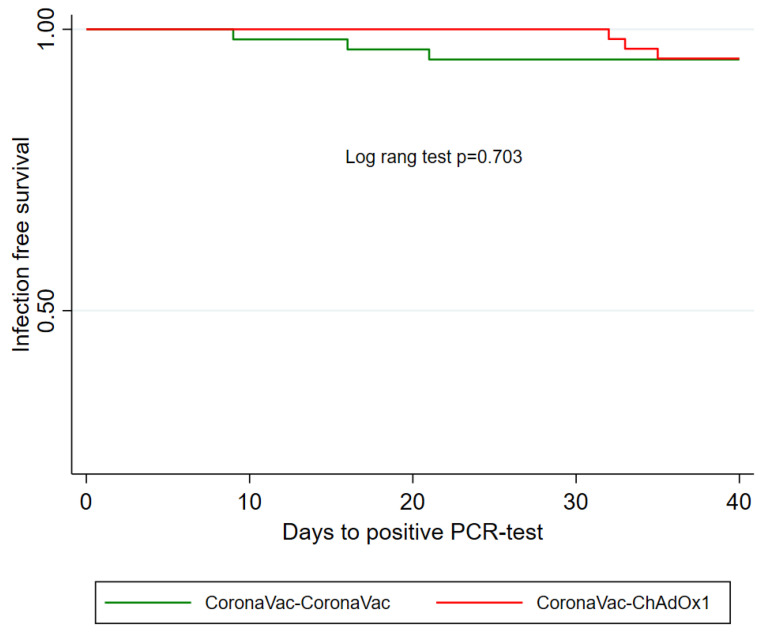
The effectiveness of COVID-19 vaccination against COVID-19 infection after 14 days of second dose injection, as assessed using the log-rank test.

The incidence of positive cases and hospitalization of Delta sub-variants COVID-19, which were collected from the 14th day of the second vaccination until the 40th day, are presented in Table 2 and Figure 2. There was no patient who was admitted to the ICU, had died, or needed mechanical ventilation after diagnosis of a breakthrough infection. Moreover, the results showed less risk of hospitalization within 40 days in people who received the heterologous CoronaVac–ChAdOx1 regimen compared with the homologous CoronaVac–CoronaVac regimen (aHR 0.13, 95% CI 0.02 to 0.82, *p* = 0.030) (Table 2).

In addition, the CoronaVac–ChAdOx1 regimen was related to a significantly lower rate of AEFIs compared with the CoronaVac–CoronaVac regimen (aHR 0.42, 95% CI 0.32 to 0.54; *p* = 0.001). In particular, the CoronaVac–ChAdOx1 regimen was independently associated with a lower frequency of any local reactions (aHR 0.55, 95% CI 0.32 to 0.93, *p* = 0.027) and a lower frequency of any systemic reactions (aHR 0.29, 95% CI 0.20 to 0.41, *p* = 0.001) compared with the CoronaVac prime and boost regimen. Table 3 shows the association between AEs and COVID-19 vaccination after IPW propensity scoring.

Participants who received the homologous CoronaVac booster dose reported significantly more systemic side effects, such as headache (4.41 per 1000 persons), myalgia (6.07 per 1000 persons), and nausea and vomiting (1.31 per 1000 persons). For local reactions, the majority of side effects were pain, swelling, and redness at the injection site (Table 3). A slightly higher frequency of AESIs was observed in people who received the homologous CoronaVac–CoronaVac regimen compared with the heterologous CoronaVac–ChAdOx1 regimen; the higher rates included paraesthesia, chest tightness, and palpitation (Table 4). There was no significance in the severity of all reactions. Four participants with palpitations had normal EKGs and the symptoms resolved after the administration of sublingual isosorbide dinitrate. One participant had an anaphylactic reaction and was successfully treated with adrenaline (Table 4).

### 3.3. Risk Factors Associated with AEFIs

The results of the univariable analysis indicated that age ≥ 50 years, male, and CoronaVac–ChAdOx1 regimen were independent risk factors associated with fewer AEFIs. In contrast, a history of drug allergy, hypertension, dyslipidaemia, diabetes mellitus, allergic rhinitis, and asthma were independent risk factors associated with higher AEFIs among the population receiving the COVID-19 vaccination (Table 5). Using multivariable analysis, the regimen of CoronaVac–ChAdOx1 was associated with fewer AEFIs than CoronaVac–CoronaVac (aHR 0.39, 95% CI 0.30 to 0.49, *p* = 0.001) after adjusting the propensity score, which included age, body weight, hypertension, diabetes mellitus, history of drug allergy, and allergic rhinitis.

**Figure 3:**
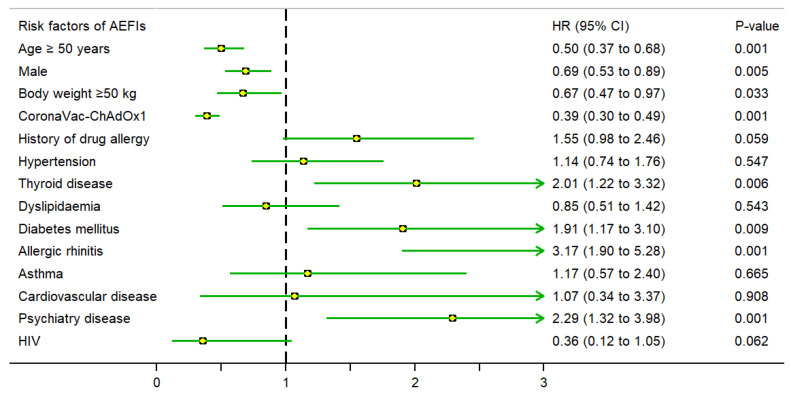
Hazard ratios for AEFIs after COVID-19 vaccination, as assessed using the multivariate Cox proportional hazards model (adjusted for propensity score, which included body weight, age, diabetes mellitus, hypertension, history of drug allergy, and allergic rhinitis).

**Figure 4:**
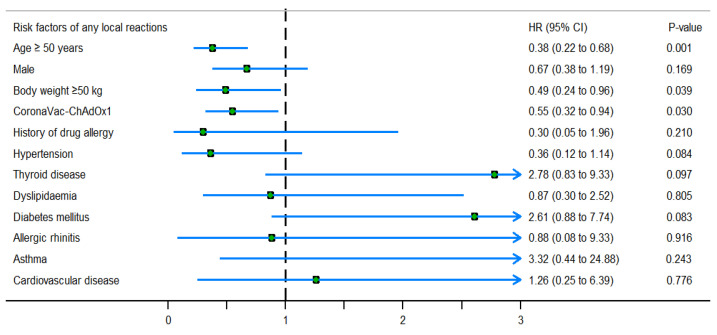
Hazard ratio for any local reactions after COVID-19 vaccination, as assessed using the multivariate Cox proportional hazards model (adjusted for propensity score, which included body weight, age, diabetes mellitus, hypertension, history of drug allergy, and allergic rhinitis).

**Figure 5:**
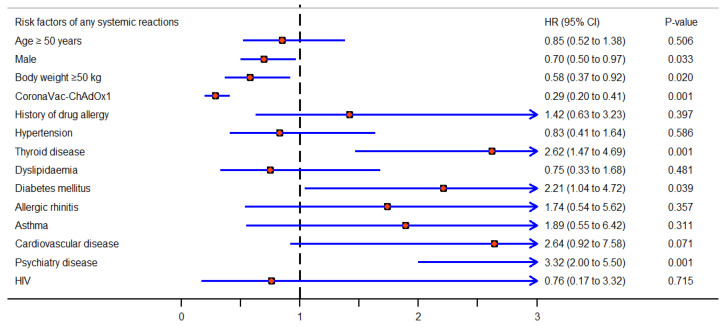
Hazard ratio for any systemic reactions after COVID-19 vaccination, as assessed using the multivariate Cox proportional hazards model (adjusted for propensity score, which included body weight, age, diabetes mellitus, hypertension, history of drug allergy, and allergic rhinitis).

Moreover, Figure 3 shows the hazard ratio for AEFIs due to COVID-19 vaccination classified by risk factors. Similarly, Figure 4 and Figure 5 present subgroup analyses of the local adverse reactions and systemic adverse reactions due to COVID-19 vaccination, respectively.

## 4. Discussion

This study mainly evaluated the effectiveness and safety of two different regimens of COVID-19 inoculation: CoronaVac–ChAdOx1 compared with CoronaVac prime and CoronaVac booster doses. The results indicate that the heterologous CoronaVac–ChAdOx1 regimen was safe, well-tolerated, and effective. Most participants reported only minor adverse effects. Additionally, the results show that independent factors associated with decreased AEFIs were age ≥ 50 years, male gender, body weight ≥ 50 kg, and the CoronaVac–ChAdOx1 regimen, while independent factors associated with increased AEFIs were thyroid disease, diabetes mellitus, allergic rhinitis, and psychiatry disease.

Recent evidence showed that the delta variant of SARS-CoV-2 could escape immune protection and diminish the efficacy of the current regimens (two doses of the same vaccine type) [3,12,13]. Therefore, several studies suggested that heterologous types of vaccines could be a solution for this as a variety of vaccine regimens could increase immune response when administered as boosters [8,13,14].

Based on the previous studies, together with the spreading of the Delta variant, the Ministry of Public Health of Thailand highly encouraged people to be inoculated with a mixing regimen of CoronaVac–ChAdOx1 because the regimen of CoronaVac only provided a 51% efficacy rate for the Delta variant [15]. However, the safety profile of the CoronaVac–ChAdOx1 regimen needs to be further studied, especially in a larger population around the world. The minor adverse reactions in our study were mostly caused by vaccine reactogenicity, which relates to pyrogenic cytokines such as IL-1, IL-6, PGE2, and TNF-α [16]. There was no severe adverse event in either the CoronaVac or ChAdOx1 receivers, except in one case, who was suspected to have anaphylaxis from the second dose of the CoronaVac vaccine. In general, the adverse event profile was similar to the previous literature on inactivated and adenoviral-vectored COVID-19 vaccines [1,14]. This study found a little lower rate of adverse events than other studies [12,17], but further comparative studies are needed to confirm these findings.

Our study found that the breakthrough infection of COVID-19 from the 14th day of the second vaccination until the 40th day was 0.69% in people who received the heterologous CoronaVac–ChAdOx1 regimen and 0.67% for the homologous CoronaVac–CoronaVac regimen. The clinical efficacy of both regimens of COVID-19 vaccine, i.e., homologous and heterologous, was promising. Patients who received either of the regimens had less severe symptoms in terms of mechanical ventilation needs and ICU admission. Moreover, this study showed a possible effect of the CoronaVac–ChAdOx1 regimen on the reduction of the hospitalization rate of people due to COVID-19 infection. The phase III trials showed that ChAdOx1 was 76% [6] effective and CoronaVac was 50.8% [18] effective at preventing symptomatic infection, and both vaccines were 100% effective at preventing serious illnesses [6,18]. The efficacy in the real world may differ from the trials due to the heterogeneity of the people who got the vaccines. Moreover, the studies were conducted in different settings and with limitations. Therefore, the vaccine efficacy in this study was much higher than in the published data.

Although the efficacy of vaccines could not be directly compared due to the differences in characteristics of the population, infectious agents, and laboratory methods, the results from real clinical settings were considered useful for the determination of the inferiority of each vaccine in terms of effectiveness. In Thailand, the Delta wave has occurred since August 2021, and the current study was conducted during this period; therefore, the results help to determine whether the Thai population should receive CoronaVac or ChAdOx1 as the second dose of vaccine.

Focusing on the risk factors of adverse events, a study indicated that people who received heterologous COVID-19 vaccination were prone to have a higher incidence of common vaccination-related adverse effects, such as fever [19]. The results of this study show no severe adverse effects and the mild adverse effects were similar to those reported in homologous COVID-19 vaccination regimens. However, the patients who received a heterologous COVID-19 vaccination were more likely to experience common vaccination-related adverse effects, such as pain, swelling, redness, and fever. In contrast with the previous studies [17,20], the current study showed that the heterologous CoronaVac–ChAdOx1 regimen was related to fewer AEFIs when compared with the homologous CoronaVac–CoronaVac regimen. Both local and systemic AEs were reported more with the CoronaVac–CoronaVac regimen in the current study. This difference might result from population-dependent vaccine effects, vaccination regimens, vaccine administration, and handling practices [21]. Moreover, several differences in study design, e.g., randomized controlled trial vs. observational study, immunization interval, and study population demographics could explain this disparity [12].

Apart from different types of vaccines, the current study summarized the factors correlated with fewer and more AEFIs. These conformed to a prospective observational study in India [22] that studied people who received two doses of ChAdOx1. The results described several risk factors associated with an increase in AEFIs, including age < 40 years (adjusted odd ratio: aOR 1.40, *p* < 0.05), female gender (aOR 1.80, *p* < 0.001), hypothyroidism (aOR 2.76, *p* = 0.04), and hypertension (aOR 1.96, *p* = 0.02). Another study also reported female gender as a risk factor for systemic AEFIs following COVID-19 vaccination [23].

A possible hypothesis that might explain the effect of gender on AEFIs was hormones that regulate cytokine levels and the immunological response to vaccination. It was shown that women developed stronger neutralizing titres after vaccination than men. The higher immune response in females might result in a more severe response to the vaccines than in males [23,24,25]. More severe local and systemic adverse effects were reported in women who received trivalent inactivated seasonal influenza vaccination (TIV) [26], as well as most other pathogen vaccines [27]. In contrast, the level of testosterone was inversely related to TIV antibody titres, and therefore caused more doses of TIV for men to achieve the same titre as women [28,29]. Laboratory results additionally indicated that female B cells produced higher antigen-specific IgG [30], and thus, the difference between the sexes in terms of the immune response due to COVID-19 vaccines was proposed [31].

Regarding the correlation between age and AEFIs, many studies suggested that younger people tended to have more AEFIs than older people due to stronger immunological responses [24,25]. Studies also provided evidence showing low levels of CRP, IL-10, and IL-6 cytokines in the elderly were lower, resulting in fewer systemic side effects [23]. For body weight, the hypothesis was high fat in the body reduced adiponectin levels. Adiponectin is an adipocyte hormone that was demonstrated to reduce macrophage activation and the production of pro-inflammatory cytokines such as TNF, IL-6, and NFkB [32].

Interestingly, there was an association between thyroid disorders and immunization found in several studies [33,34]. A proposed hypothesis of this phenomenon was that COVID-19 vaccination increased blood viscosity, leading to hyperviscosity [34]. Furthermore, hyperviscosity caused an abnormally high thyroid hormone level [35]. There was a case report that mentioned a female patient who acquired sub-acute thyroiditis shortly after receiving the adenovirus-vectored COVID-19 (ChAdOx1) vaccine [36]. Likewise, another case report of sub-acute thyroiditis after inactive SARS-CoV-2 virus (CoronaVac) vaccination was published [37]. However, further studies on thyroid function in healthy people and thyroid patients who received the COVID-19 vaccine are needed [38].

Focusing on the allergic profile of the vaccines, CoronaVac, which contains the entire inactivated virus, aluminum hydroxide adjuvant [20], and some mineral salts, was reported to cause urticaria in approximately 0.8% of vaccine receivers in Turkey [39,40]. The US Center for Disease Control and Prevention (CDC) recommended that anyone who experiences an immediate allergic response within 4 h of vaccination should not receive the same vaccine again [41], and thus, heterologous types of vaccines, such as the CoronaVac–ChAdOx1 regimen could be an alternative for such people. Although the effectiveness and safety of vaccine regimens in this study were not compared with unvaccinated people due to ethical reasons and others, the findings in this study were consistent with previous studies and enough to advise the use of heterologous vaccines in people if needed [42].

There were some limitations in this study that should be noted. The difference in many baseline characteristics between the two groups was observed and might cause different results. Although the IPW propensity score method and multivariable Cox regression analysis were used to adjust this difference, all readers should bear in mind that the results were from two groups of people who might have some dissimilar backgrounds. In addition, the study was conducted in only one centre, and thus, the characteristics of the population in this study might not be the same as in other centres or other areas. Interpretation of the results should be cautious in case other people have different reactogenicity to the vaccines. Because immunogenicity was not measured in this study, it could not be concluded that people recruited in this area had the same immune response as others. Moreover, this study primarily focused on the safety aspects of different vaccine regimens. While it meticulously evaluated vaccine safety and adverse events following immunization (AEFIs), it did not include an assessment of vaccine efficacy in terms of antibody titration and neutralization efficacy. This aspect should be considered in future research. In addition, the focus of this study on the homologous regimen consisting of two doses of CoronaVac only was one of the limitations; the homologous regimen involving two doses of ChAdOx1 was not investigated in this study. However, the homologous regimen involving two doses of ChAdOx1 was not investigated. The decision to explore the homologous regimen with two doses of ChAdOx1 holds research value due to several significant considerations. While the ChAdOx1 vaccine showed promising efficacy and immunogenicity in clinical trials, the investigation of its safety and effectiveness in a real-world setting remains pivotal. Understanding the real-world performance of the ChAdOx1 vaccine in a homologous regimen contributes to evidence-based decision-making in public health strategies. Lastly, regarding sample size, this study was considered a small study with less than a thousand participants, although this study included the largest number of people who received the heterologous CoronaVac–ChAdOx1 regimen in Thailand. Studies in the future should collect data from more people, especially at the time when more vaccines were available in Thailand. Moreover, the safety and efficacy of COVID-19 vaccines in special populations, including children, older patients, pregnant women, and lactating mothers, remain essential areas of study. While this study primarily focused on adult populations, future research should investigate the performance of different vaccination regimens in these vulnerable groups to ensure comprehensive protection against COVID-19. However, the ongoing evolution of the virus and the emergence of new variants underscores the need for continued vaccine development. Research into vaccines that offer broad protection against a range of SARS-CoV-2 variants should also be emphasized. Additionally, exploring novel vaccine platforms and technologies may provide innovative solutions for future pandemics.

## 5. Conclusions

This study found that a heterologous schedule of CoronaVac–ChAdOx1 was effective in preventing symptomatic COVID-19 with higher effectiveness against hospitalization mainly due to the Delta variant. Moreover, the administration of the CoronaVac vaccine first and the ChAdOx1 nCoV-19 vaccine 3 weeks after could reduce vaccine-associated adverse effects. The risk factors that produced fewer AEFIs included age ≥ 50 years, male gender, and body weight ≥ 50 kg. Thyroid disease, diabetes mellitus, allergic rhinitis, and psychiatric disorders were the risk factors associated with an increase in AEFIs. The government immunization programme should consider the implementation of the heterologous schedule of CoronaVac–ChAdOx1, especially for individuals who have previously experienced AEFIs with other vaccine doses and during the period of Delta variant COVID-19. The findings in this study underscore the significance of incorporating heterologous vaccination regimens as a primary option for the government immunization programme.

## Figures and Tables

**Table 1:** Demographic and clinical characteristics of population who received COVID-19 vaccines.

Characteristics	CoronaVac–CoronaVac (n = 430)	CoronaVac–ChAdOx1 (n = 445)	*p*-Value
Sex, n (%)			
Male	219 (50.93)	227 (51.01)	1.000
Female	211 (49.07)	218 (48.99)	
Age, years, mean ± SD	40.20 ± 10.01	41.83 ± 14.03	0.049
Body weight, kg, mean ± SD	66.95 ± 13.96	64.43 ± 13.60	0.007
38–49 kg	37 (8.60)	45 (10.11)	0.487
≥50 kg	393 (91.40)	340 (89.89)	
Comorbidities *, n (%)			
Hypertension	38 (8.84)	69 (15.51)	0.003
Dyslipidaemia	39 (9.07)	38 (8.54)	0.812
Thyroid disease	6 (1.40)	7 (1.57)	1.000
Cardiovascular disease	4 (0.93)	4 (0.90)	1.000
Diabetes mellitus	13 (3.02)	23 (5.17)	0.127
Chronic kidney disease	1(0.23)	5 (1.12)	0.218
Asthma	3 (0.70)	2 (0.45)	0.682
Cancer	1 (0.23)	2 (0.45)	1.000
Chronic liver disease	0 (0.00)	2 (0.45)	0.500
Allergic rhinitis	8 (1.86)	1 (0.22)	0.019
History of drug allergy	16 (3.72)	7 (1.57)	0.057
Psychiatry	5 (1.16)	3 (0.67)	0.499
HIV	2 (0.47)	1 (0.22)	0.618
Gout	4 (0.93)	3 (0.67)	0.721
Mean (SD) interval between 1st and 2nd dose (days)	21.30 ± 1.00	21.04 ± 0.29	0.001

* One person might have had more than one comorbidity. The comparisons across two COVID-19 vaccine regimens were performed using the chi-squared test or Fisher’s exact test (if less than five observations) for categorical data, and an independent *t*-test for continuous data.

**Table 2:** Incidence of people with COVID-19 positive and hospitalization due to COVID-19 within 40 days of second dose of COVID-19 vaccination.

Event	No. Reporting (Incidence Rate *)		Crude HR ** (95% CI)	*p*-Value	Adjusted HR *** (95% CI)	*p*-Value
	CoronaVac–CoronaVac (n = 430)	CoronaVac–ChAdOx1 (n = 445)				
COVID-19 infection	3 (1.7)	3 (1.6)	0.96 (0.19–4.77)	0.963	2.24 (0.46–10.90)	0.317
Hospitalization due to COVID-19 infection	1 (0.58)	1 (0.56)	0.96 (0.06–15.40)	0.979	0.13 (0.02–0.82)	0.030

Crude HR, crude hazard ratio; adjusted HR, adjusted hazard ratio. * Incidence rate is a crude incidence expressed as an event per 10,000 persons. ** Univariate Cox proportional hazards model; *** multivariate cox proportional hazards model (adjusted for propensity score, which included body weight, hypertension, age, history of drug allergy, and allergic rhinitis).

**Table 3:** Frequency and hazard ratio of the adverse events reported by people that received different regimens of COVID-19 vaccine.

Event	No. Reporting AEFIs (Incidence Rate *)		Crude HR ** (95% CI)	*p*-Value	Adjusted HR *** (95% CI)	*p*-Value
	CoronaVac–CoronaVac (n = 430)	CoronaVac–ChAdOx1 (n = 445)				
Total AEFIs	152 (18.09)	72 (6.43)	0.43 (0.33–0.57)	0.001	0.42 (0.32–0.54)	0.001
Many local reactions	36 (4.29)	23 (2.05)	0.58 (0.35–0.99)	0.047	0.55 (0.32–0.93)	0.027
Rash	6 (0.71)	8 (0.23)	1.22 (0.42–3.53)	0.705	1.14 (0.38–3.42)	0.816
Pain	27 (3.21)	54 (4.83)	1.83 (1.15- 2.91)	0.010	1.71 (1.09–2.68)	0.019
Swelling	27 (3.21)	49 (4.83)	1.66 (1.03–2.66)	0.034	1.54 (0.98–2.43)	0.063
Redness	27 (3.21)	49 (4.83)	1.66 (1.03–2.66)	0.034	1.54 (0.98–2.43)	0.063
Many systemic reactions	107 (12.74)	36 (3.22)	0.31 (0.21–0.45)	0.001	0.29 (0.20–0.41)	0.001
Fever	15 (1.79)	30 (2.68)	1.82 (0.98–3.39)	0.057	1.57 (0.86–2.89)	0.139
Headache	37 (4.41)	17 (1.52)	0.42 (0.23–0.74)	0.003	0.36 (0.20–0.64)	0.001
Fatigue	11 (1.31)	20 (1.78)	1.65 (0.79–3.46)	0.177	1.61 (0.77–3.36)	0.208
Nausea/vomiting	11 (1.31)	7 (0.63)	0.59 (0.23–1.51)	0.268	0.38 (0.15–0.98)	0.045
Diarrhea	9 (1.07)	8 (0.72)	0.81 (0.31–2.08)	0.656	0.86 (0.32–2.32)	0.774
Myalgia	51 (6.07)	42 (3.75)	0.75 (0.50–1.13)	0.176	0.61 (0.40–0.94)	0.001

Note: One person might have had more than one event. Incidence was reported within 30 days since any of the two doses. Crude HR, crude hazard ratio; adjusted HR, adjusted hazard ratio. * Incidence rate is a crude incidence expressed as an event per 1000 persons. ** Univariate Cox proportional hazards model; *** multivariate Cox proportional hazards model (adjusted for propensity score, which included body weight, age, diabetes mellitus, hypertension, history of drug allergy, and allergic rhinitis).

**Table 4:** Adverse events of special interest (AESIs) that were reported within 30 days after two doses of vaccines.

Adverse Reaction	Event (%)		*p*-Value *
	CoronaVac–CoronaVac (n = 430)	CoronaVac–ChAdOx1 (n = 445)	
Participants with any AESI	12 (2.79)	3 (0.67)	0.018
*Anaphylaxis*			
Anaphylactic reaction	1 (0.23)	0 (0)	0.491
*Neurologic disorders*			
Paraesthesia	3 (0.70)	1 (0.22)	0.366
*Cardiac disorders*			
Palpitation	4 (0.93)	0 (0)	0.059
*Eye disorders*			
Blurred vision	0 (0)	1 (0.22)	1.000
*Respiratory, thoracic, and mediastinal disorders*			
Shortness of breath	1 (0.23)	1 (0.22)	1.000
Chest tightness	3 (0.36)	0 (0.00)	0.118

* Fisher’s exact test.

**Table 5:** Univariate and multivariate analyses identifying risk factors of AEFIs among all of the population with COVID-19 vaccination.

Variable	Crude HR * (95% CI)	*p*-Value	Adjusted HR ** (95% CI)	*p*-Value
Risk factors of AEFIs				
Age ≥ 50 years	0.55 (0.40 to 0.74)	0.001	0.50 (0.37 to 0.68)	0.001
Male	0.62 (0.48 to 0.82)	0.001	0.69 (0.53 to 0.89)	0.005
Body weight ≥ 50 kg	0.71 (0.47 to 1.05)	0.084	0.67 (0.47 to 0.97)	0.033
CoronaVac–ChAdOx1	0.43 (0.33 to 0.58)	0.001	0.39 (0.30 to 0.49)	0.001
History of drug allergy	2.34 (1.31 to 4.18)	0.004	1.55 (0.98 to 2.45)	0.059
Hypertension	1.57 (1.12 to 2.21)	0.010	1.14 (0.74 to 1.75)	0.547
Thyroid disease	2.11 (0.99 to 4.48)	0.051	2.00 (1.21 to 3.32)	0.006
Dyslipidaemia	1.69 (1.16 to 2.48)	0.006	0.85 (0.51 to 1.42)	0.543
Diabetes mellitus	2.31 (1.46 to 3.65)	0.001	1.91 (1.17 to 3.09)	0.009
Allergic rhinitis	4.43 (2.19 to 8.98)	0.001	3.16 (1.90 to 5.28)	0.001
Asthma	3.13 (1.17 to 8.43)	0.024	1.17 (0.57 to 2.40)	0.665
Cardiovascular disease	1.94 (0.72 to 5.21)	0.190	1.07 (0.34 to 3.37)	0.908
Psychiatric disease	1.94 (0.72 to 5.21)	0.190	2.29 (1.32 to 3.98)	0.003
HIV	1.28 (0.18 to 9.10)	0.808	0.36 (0.12 to 1.05)	0.062

Crude HR, crude hazard ratio; adjusted HR, adjusted hazard ratio. * Univariate Cox proportional hazards model; ** multivariate Cox proportional hazards model (adjusted for propensity score, which included body weight, age, diabetes mellitus, hypertension, history of drug allergy, and allergic rhinitis).

## Data Availability

The datasets used and analyzed during the current study are available from the corresponding author upon reasonable request.

## References

[1] Duarte-Salles T., Prieto-Alhambra D. (2021). Heterologous vaccine regimens against COVID-19. Lancet.

[2] Sharma K., Koirala A., Nicolopoulos K., Chiu C., Wood N., Britton P.N. (2021). Vaccines for COVID-19: Where do we stand in 2021?. Paediatr. Respir. Rev..

[3] Jara A., Undurraga E.A., González C., Paredes F., Fontecilla T., Jara G., Pizarro A., Acevedo J., Leo K., Leon F. (2021). Effectiveness of an inactivated SARS-CoV-2 Vaccine in Chile. N. Engl. J. Med..

[4] Ramasamy M.N., Minassian A.M., Ewer K.J., Flaxman A.L., Folegatti P.M., Owens D.R., Voysey M., Aley P.K., Angus B., Babbage G. (2021). Safety and immunogenicity of ChAdOx1 nCoV-19 vaccine administered in a prime-boost regimen in young and old adults (COV002): A single-blind, randomised, controlled, phase 2/3 trial. Lancet.

[5] Falsey A.R., Sobieszczyk M.E., Hirsch I., Sproule S., Robb M.L., Corey L., Neuzil K.M., Hahn W., Hunt J., Mulligan M.J. (2021). Phase 3 Safety and Efficacy of AZD1222 (ChAdOx1 nCoV-19) COVID-19 Vaccine. N. Engl. J. Med..

[6] Voysey M., Costa Clemens S.A., Madhi S.A., Weckx L.Y., Folegatti P.M., Aley P.K., Angus B., Baillie V.L., Barnabas S.L., Bhorat Q.E. (2021). Single-dose administration and the influence of the timing of the booster dose on immunogenicity and efficacy of ChAdOx1 nCoV-19 (AZD1222) vaccine: A pooled analysis of four randomised trials. Lancet.

[7] Department of Disease Control (2021). COVID-19 Vaccination Guideline under Pandemic B.E.2564, Thailand. https://www.ddc.moph.go.th/uploads/files/1729520210301021023.pdf.

[8] Yorsaeng R., Vichaiwattana P., Klinfueng S., Wongsrisang L., Sudhinaraset N., Vongpunsawad S., Poovorawan Y. (2021). Immune response Elicited from Heterologous SARS-CoV-2 Vaccination: Sinovac (CoronaVac) Followed by AstraZeneca (Vaxzevria). medRxiv.

[9] Wanlapakorn N., Suntronwong N., Phowatthanasathian H., Yorsaeng R., Vichaiwattana P., Thongmee T., Auphimai C., Srimuan D., Thatsanatorn T., Assawakosri S. (2022). Safety and immunogenicity of heterologous and homologous inactivated and adenoviral-vectored COVID-19 vaccine regimens in healthy adults: A prospective cohort study. Hum. Vaccines Immunother..

[10] Niyomnaitham S., Toh Z.Q., Wongprompitak P., Jansarikit L., Srisutthisamphan K., Sapsutthipas S., Jantraphakorn Y., Mingngamsup N., Licciardi P.V., Chokephaibulkit K. (2022). Immunogenicity and reactogenicity against the SARS-CoV-2 variants following heterologous primary series involving CoronaVac, ChAdox1 nCov-19 and BNT162b2 plus BNT162b2 booster vac-cination: An open-label randomized study in healthy Thai adults. Hum. Vaccines Immunother..

[11] Mahasirimongkol S., Khunphon A., Kwangsukstid O., Sapsutthipas S., Wichaidit M., Rojanawiwat A., Wichuckchinda N., Puangtubtim W., Pimpapai W., Soonthorncharttrawat S. (2022). The Pilot Study of Immunogenicity and Adverse Events of a COVID-19 Vaccine Regimen: Priming with Inactivated Whole SARS-CoV-2 Vaccine (CoronaVac) and Boosting with the Adenoviral Vector (ChAdOx1 nCoV-19) Vaccine. Vaccines.

[12] Hillus D., Schwarz T., Tober-Lau P., Vanshylla K., Hastor H., Thibeault C., Jentzsch S., Helbig E.T., Lippert L.J., Tscheak P. (2021). Safety, reactogenicity, and immunogenicity of homologous and heterologous prime-boost immunisation with ChA-dOx1 nCoV-19 and BNT162b2: A prospective cohort study. Lancet Respir. Med..

[13] Wanlapakorn N., Suntronwong N., Phowatthanasathian H., Yorsaeng R., Thongmee T., Vichaiwattana P., Auphimai C., Wongsrisang L., Klinfueng S., Sudhinaraset N. (2022). Immunogenicity of heterologous inactivated and adenoviral-vectored COVID-19 vaccine: Real-world data. Vaccine.

[14] Niyomnaitham S., Sewatanon J., Senawong S., Angkasekwinai N., Sirilak S., Uppapong B. Safety and Immunological Re-sponse Following Heterologous Primary Series of COVID-19 Vaccination: The Preliminary Report Focusing on the Delta Var-iant. https://sicres.org/wp-content/uploads/2021/08/Poster-hetero-in-Eng_8pm.pdf.

[15] Palacios R., Batista A.P., Nascimento C.S., Patiño E.G., Santos J.P., Tilli Reis P.C. Efficacy and Safety of a COVID-19 Inac-tivated Vaccine in Healthcare Professionals in Brazil: The PROFISCOV Study. https://ssrn.com/abstract=3822780.

[16] Parajuli S.B., Shakya A., Koirala S.B., KC H., Koirala P. (2021). Adverse events following immunisation after COVISHIELD vac-cination among Nepali population of eastern Nepal. J. Patan Acad. Health Sci..

[17] Folegatti P.M., Ewer K.J., Aley P.K., Angus B., Becker S., Belij-Rammerstorfer S., Bellamy D., Bibi S., Bittaye M., Clutter-buck E.A. (2020). Safety and immunogenicity of the ChAdOx1 nCoV-19 vaccine against SARS-CoV-2: A preliminary report of a phase 1/2, single-blind, randomised controlled trial. Lancet.

[18] Kim J.H., Marks F., Clemens J.D. (2021). Looking beyond COVID-19 vaccine phase 3 trials. Nat. Med..

[19] Shaw R.H., Stuart A., Greenland M., Liu X., Nguyen Van-Tam J.S., Snape M.D., Com-COV Study Group (2021). Heterologous prime-boost COVID-19 vaccination: Initial reactogenicity data. Lancet.

[20] Zhang Y., Zeng G., Pan H., Li C., Hu Y., Chu K., Han W., Chen Z., Tang R., Yin W. (2021). Safety, tolerability, and im-munogenicity of an inactivated SARS-CoV-2 vaccine in healthy adults aged 18-59 years: A randomised, double-blind, placebo-controlled, phase 1/2 clinical trial. Lancet Infect. Dis..

[21] Tregoning J.S., Flight K.E., Higham S.L., Wang Z., Pierce B.F. (2021). Progress of the COVID-19 vaccine effort: Viruses, vaccines and variants versus efficacy, effectiveness and escape. Nat. Rev. Immunol..

[22] Kaur U., Ojha B., Pathak B.K., Singh A., Giri K.R., Singh A., Das A., Misra A., Yadav A.K., Kansal S. (2021). A prospective observational safety study on ChAdOx1 nCoV-19 corona virus vaccine (recombinant) use in healthcare workers-first results from India. EClinicalMedicine.

[23] Joshi R.K., Muralidharan C.G., Gulati D.S., Mopagar V., Dev J.K., Kuthe S., Rather A.A., Sahoo A.K. (2021). Higher incidence of reported adverse events following immunisation (AEFI) after first dose of COVID-19 vaccine among previously infected health care workers. Med. J. Armed Forces India.

[24] Jayadevan R., Shenoy R., Anithadevi T.S. (2021). Survey of Symptoms Following COVID-19 Vaccination in India. medRxiv.

[25] Potluri T., Fink A.L., Sylvia K.E., Dhakal S., Vermillion M.S., Vom Steeg L., Deshpande S., Narasimhan H., Klein S.L. (2019). Age-associated changes in the impact of sex steroids on influenza vaccine responses in males and females. NPJ Vaccines.

[26] Flanagan K.L., Fink A.L., Plebanski M., Klein S.L. (2017). Sex and Gender Differences in the Outcomes of Vaccination over the Life Course. Annu. Rev. Cell Dev. Biol..

[27] Klein S.L., Marriott I., Fish E.N. (2015). Sex-based differences in immune function and responses to vaccination. Trans. R. Soc. Trop. Med. Hyg..

[28] Engler R.J., Nelson M.R., Klote M.M., VanRaden M.J., Huang C.Y., Cox N.J., Klimov A., Keitel W.A., Nichol K.L., Carr W.W. (2008). Half- vs. full-dose trivalent inactivated influenza vaccine (2004–2005): Age, dose, and sex effects on immune responses. Arch. Intern. Med..

[29] Furman D., Hejblum B.P., Simon N., Jojic V., Dekker C.L., Thiébaut R., Tibshirani R.J., Davis M.M. (2014). Systems analysis of sex differences reveals an immunosuppressive role for testosterone in the response to influenza vaccination. Proc. Natl. Acad. Sci. USA.

[30] Voigt E.A., Ovsyannikova I.G., Kennedy R.B., Grill D.E., Goergen K.M., Schaid D.J., Poland G.A. (2019). Sex differences in older adults’ immune responses to seasonal influenza vaccination. Front. Immunol..

[31] Takahashi T., Ellingson M.K., Wong P., Israelow B., Lucas C., Klein J., Silva J., Mao T., Oh J.E., Tokuyama M. (2020). Sex differences in immune responses that underlie COVID-19 disease outcomes. Nature.

[32] Klöting N., Blüher M. (2014). Adipocyte dysfunction, inflammation and metabolic syndrome. Rev. Endocr. Metab. Disord..

[33] Vera-Lastra O., Ordinola Navarro A., Cruz Domiguez M.P., Medina G., Sánchez Valadez T.I., Jara L.J. (2021). Two cases of Graves’ disease following SARS-CoV-2 vaccination: An autoimmune/inflammatory syndrome induced by adjuvants. Thyroid.

[34] Joob B., Wiwanitkit V. (2021). Expected viscosity after COVID-19 vaccination, hyperviscosity and previous COVID-19. Clin. Appl. Thromb. Hemost..

[35] Tamagna E., Hershman J., Premachandra B.N. (1979). Circulating thyroid hormones in a patient with hyperviscosity syndrome. Clin. Chim. Acta.

[36] Oyibo S.O. (2021). Subacute Thyroiditis After Receiving the Adenovirus-Vectored Vaccine for Coronavirus Disease (COVID-19). Cureus.

[37] Saygılı E.S., Karakilic E. (2021). Subacute thyroiditis after inactive SARS-CoV-2 vaccine. BMJ Case Rep..

[38] Mungmunpuntipantip R., Wiwanitkit V. (2021). Abnormal Thyroid Function following COVID-19 Vaccination. Indian J. Endocrinol. Metab..

[39] Riad A., Sağıroğlu D., Üstün B., Pokorná A., Klugarová J., Attia S., Klugar M. (2021). Prevalence and Risk Factors of CoronaVac Side Effects:An Independent Cross-Sectional Study among Healthcare Workers in Turkey. J. Clin. Med..

[40] Laisuan W., Wongsa C., Chiewchalermsri C., Thongngarm T., Rerkpattanapipat T., Iamrahong P., Ruangwattanachok C., Nanthapisal S., Sompornrattanaphan M. (2021). CoronaVac COVID-19 Vaccine-Induced Anaphylaxis: Clinical Characteristics and Revaccination Outcomes. J. Asthma Allergy.

[41] CDC COVID-19 Vaccines and Severe Allergic Reactions. https://www.cdc.gov/coronavirus/2019-ncov/vaccines/safety/allergic-reaction.html.

[42] Dhanda S., Osborne V., Lynn E., Shakir S. (2022). Postmarketing studies: Can they provide a safety net for COVID-19 vaccines in the UK?. BMJ Evid. Based Med..

